# Whole body FDG PET/MR for progression free and overall survival prediction in patients with relapsed/refractory large B-cell lymphomas undergoing CAR T-cell therapy

**DOI:** 10.1186/s40644-022-00513-y

**Published:** 2022-12-27

**Authors:** Therese Sjöholm, Alexander Korenyushkin, Gustav Gammelgård, Tina Sarén, Tanja Lövgren, Angelica Loskog, Magnus Essand, Joel Kullberg, Gunilla Enblad, Håkan Ahlström

**Affiliations:** 1 Department of Surgical Sciences, Uppsala University, Uppsala, Sweden; 2 Antaros Medical AB, Mölndal, Sweden; 3 Department of Immunology, Genetics and Pathology, Uppsala University, Uppsala, Sweden

**Keywords:** PET/MR, ADC, FDG, LBCL, CAR T-cells, Bone marrow, Immunotherapy

## Abstract

**Background:**

To find semi-quantitative and quantitative Positron Emission Tomography/Magnetic Resonance (PET/MR) imaging metrics of both tumor and non-malignant lymphoid tissue (bone marrow and spleen) for Progression Free Survival (PFS) and Overall Survival (OS) prediction in patients with relapsed/refractory (r/r) large B-cell lymphoma (LBCL) undergoing Chimeric Antigen Receptor (CAR) T-cell therapy.

**Methods:**

A single-center prospective study of 16 r/r LBCL patients undergoing CD19-targeted CAR T-cell therapy. Whole body 18F-fluorodeoxyglucose (FDG) PET/MR imaging pre-therapy and 3 weeks post-therapy were followed by manual segmentation of tumors and lymphoid tissues. Semi-quantitative and quantitative metrics were extracted, and the metric-wise rate of change (Δ) between post-therapy and pre-therapy calculated. Tumor metrics included maximum Standardized Uptake Value (SUV_max_), mean SUV (SUV_mean_), Metabolic Tumor Volume (MTV), Tumor Lesion Glycolysis (TLG), structural volume (V), total structural tumor burden (V_total_) and mean Apparent Diffusion Coefficient (ADC_mean_). For lymphoid tissues, metrics extracted were SUV_mean_, mean Fat Fraction (FF_mean_) and ADC_mean_ for bone marrow, and SUV_mean_, V and ADC_mean_ for spleen. Univariate Cox regression analysis tested the relationship between extracted metrics and PFS and OS. Survival curves were produced using Kaplan–Meier analysis and compared using the log-rank test, with the median used for dichotomization. Uncorrected *p*-values < 0.05 were considered statistically significant. Correction for multiple comparisons was performed, with a False Discovery Rate (FDR) < 0.05 considered statistically significant.

**Results:**

Pre-therapy (*p* < 0.05, FDR < 0.05) and Δ (*p* < 0.05, FDR > 0.05) total tumor burden structural and metabolic metrics were associated with PFS and/or OS. According to Kaplan-Meier analysis, a longer PFS was reached for patients with pre-therapy MTV ≤ 39.5 ml, ΔMTV≤1.35 and ΔTLG≤1.35. ΔSUV_max_ was associated with PFS (*p* < 0.05, FDR > 0.05), while ΔADC_mean_ was associated with both PFS and OS (*p* < 0.05, FDR > 0.05). ΔADC_mean_ > 0.92 gave longer PFS and OS in the Kaplan-Meier analysis. Pre-therapy bone marrow SUV_mean_ was associated with PFS (*p* < 0.05, FDR < 0.05) and OS (*p* < 0.05, FDR > 0.05). For bone marrow FDG uptake, patient stratification was possible pre-therapy (SUV_mean_ ≤ 1.8).

**Conclusions:**

MTV, tumor ADC_mean_ and FDG uptake in bone marrow unaffected by tumor infiltration are possible PET/MR parameters for prediction of PFS and OS in r/r LBCL treated with CAR T-cells.

**Trial registration:**

EudraCT 2016–004043-36.

**Supplementary Information:**

The online version contains supplementary material available at 10.1186/s40644-022-00513-y.

## Background

CD19-targeted Chimeric Antigen Receptor (CAR) T-cells have successfully been used in treatment of relapsed/refractory (r/r) large B-cell lymphomas (LBCL) with three commercially available therapies [1–3]. Although complete response rates of 40–59% have been shown in clinical trials [4], the therapy is associated with e.g. life-threatening toxicities, antigen escape and poor tumor infiltration that limit therapeutic efficacy [5]. In addition, a significant cost is currently associated with the treatment. Biomarkers that predict durable response are hence needed, either at baseline to identify patients likely to respond or early post-therapy to detect therapy failure [6].

18F-fluorodeoxyglucose (FDG) Positron Emission Tomography/Computed Tomography (PET/CT) is a recognized imaging modality for staging and response evaluation in lymphoma for standard chemo- and immunochemo-therapy treatments regimens [7–9]. In addition, FDG PET/CT is a promising tool for identifying responding/non-responding patients after CAR T-cell therapy. In patients with LBCL undergoing CAR T-cell therapy, usage of pre-therapy FDG PET tumor metrics such as the Metabolic Tumor Volume (MTV), Total Lesion Glycolysis (TLG) and maximum Standardized Uptake Value (SUV_max_) have been associated with treatment outcome [10–15]. A lower tumor burden and/or lower tumor FDG uptake have been linked to better outcomes. An early metabolic response after treatment has also proved predictive of therapy success, assessed visually [13, 16] or semi-quantitatively using SUV_max_ [12, 13, 15]. In general, the studies performed are however small and mixed results have been reported in terms of which predictive metric to use. Furthermore, to the best of our knowledge, there is no study using FDG PET/MR for CAR T-cell therapy evaluation.

Usage of FDG PET/MR in lymphoma has been shown comparable to FDG PET/CT for diagnosis [17, 18]. In addition to structural imaging, MR can provide functional tumor information without exposure to ionizing radiation. Whole-body Diffusion Weighted Imaging (DWI) [19] and subsequent quantification using the tumor Apparent Diffusion Coefficient (ADC) has been described as promising for assessing treatment response in lymphoma [20]. Previous studies of chemo- and immunochemo-therapy in non-Hodgkin lymphoma have shown that an increase in tumor ADC is detectable as early as 1–2 weeks post-therapy [21] and associated with therapy outcome [22–24].

In addition to tumor evaluations, the acquisitions of whole body imaging datasets make system wide assessments possible. Of particular interest is imaging of lymphoid tissues, e.g. bone marrow and spleen, as immunological mechanisms could play a role in the efficacy of CAR T-cell therapy [25]. Metabolic changes in lymphoid tissues have been associated with immunotherapy outcome using both PET/CT [26] and PET/MR [27] for checkpoint inhibitor therapy (CIT) in melanoma, but for CAR T-cell therapy less has been reported. Derlin et al. however showed that larger decreases in post-therapeutic FDG PET uptake in normal spleen and lymph nodes were associated with unfavorable CAR T-cell therapy outcome in a small cohort of LBCL patients (*n* = 7) scanned on PET/CT [28].

The aim of this study was to find semi-quantitative and quantitative PET/MR imaging metrics of both tumor and non-malignant lymphoid tissue (bone marrow and spleen) for PFS and OS prediction in patients with r/r LBCL undergoing CAR T-cell therapy.

## Methods

### Study design and population

This single-center prospective study was approved by the Regional Ethics Review Board and all patients gave their informed written consent to participate. Trial inclusion criteria included relapsed/refractory CD19+ B-cell lymphoma with no other curative treatment option available, measurable disease at inclusion and Eastern cooperative oncology group performance status (ECOG PS) score 0–2. Patients with a higher ECOG PS score were considered too ill to tolerate the intensive CAR T-cell therapy [29]. Exclusion criteria included pregnancy, presence of primary central nervous system lymphoma, known human immunodeficiency virus infection and an active/severe infection. The study protocol included bridging therapy of the treating clinician’s choice, with chemo- and/or radiotherapy for 4–8 weeks to reduce the tumor burden, and preconditioning with Fludarabine and Cyclophosphamide lymphodepleting therapy at 2–4 days before CAR T-cell therapy. Third generation CD19-directed CAR T-cells with CD28 and 4-1BB as co-stimulatory domains along with the CD3z signaling domain was administered as previously described [30]. A second dose of CAR T-cells was administered 4–6 weeks after the first CAR T-cell infusion if the patient did not have rapid progression or a cytokine release syndrome of ≥ grade 3.

Therapy response assessment was performed using whole body FDG PET/MR, with imaging at a minimum of two time points: pre-therapy (t_0_) and 3–4 weeks post-therapy (t_1_). Depending on clinical status, further imaging was performed 2–6 months post-therapy for a subset of patients (t_2_-t_4_).

### Imaging protocol

Whole body PET/MR imaging (head to thighs) was performed 60 min post tracer injection of FDG (3 MBq/kg injection). Patients fasted for a minimum of 6 h and were confirmed to have a blood glucose level < 10 mmol/l before tracer injection. An integrated scanner capable of simultaneous time of flight PET and 3 T MR imaging was used (Signa, GE Healthcare). Acquired whole body MR sequences included free-breathing DWI (echo time (TE)/repetition time (TR) = 62/3500 ms, inversion time (TI) = 245 ms, field of view (FOV) = 440 × 352 mm^2^, acquisition matrix = 96 × 128, slice thickness = 6 mm, b-values = 50, 400, 900 s/mm^2^) and breath-hold structural T1-weighted LAVA Dixon MR (TE/TR = 4.1/1.67 ms, flip angle 12°, FOV = 500 × 450 mm^2^, acquisition matrix 256 × 212, slice thickness 2.5 mm). Station-wise ADC maps were calculated on the scanner console using the three acquired b-values and a mono-exponential fit. Static 3 min per bed PET images were acquired and reconstructed using an iterative algorithm (Vue point FX, 2 iterations and 28 subsets, 5 mm standard Gaussian filter, FOV 500 mm, matrix 192 × 192). A vendor-provided MR Dixon-based attenuation correction was used. SUV images normalized to body weight were calculated.

### Image analysis

The Lugano classification for response assessment of lymphomas was used as reference standard [31]. Classification was performed by a Radiologist (AK) at t_1_.

#### Tumor segmentation and metric extraction

Tumors were segmented using the open source software 3DSlicer [32] by two Radiologists in consensus (AK and HA) with access to all imaging data. All visible tumors were delineated manually and separately on FDG PET, T1-weighted LAVA flex water MR and b = 900 s/mm^2^ DWI. For the DWI data, tumor tissue affected by motion between acquired b-value images was not segmented. Measurable tumors were defined according to Lugano classification as having a longest diameter of > 15 mm for nodal disease and > 10 cm for extra-nodal disease [31]. Corresponding lesions on pre- and post-therapy images were manually identified by a Radiologist (AK).

Quantitative and semi-quantitative tumor metrics were extracted with Matlab (R2021b). For each segmented tumor, SUV_mean_, SUV_max_, MTV and TLG for FDG PET, volume (V) for structural MR, and ADC_mean_ for DWI, were extracted. Whole body total tumor burden was calculated for MTV, TLG and structural volume (V_total_). A new lesion appearing at the post-therapy scan was added to the post-therapy MTV, TLG and V_total_ if its longest diameter was > 15 mm for nodal disease and > 10 cm for extra-nodal disease on T1-weighted LAVA flex water MR. For post-therapy response assessment, the rate of change (Δ) between early post-therapy and pre-therapy extracted metrics were calculated: Δ = metric (t_1_) /metric (t_0_).

For calculation of Δ, target lesions for SUV_mean_, SUV_max_, V and ADC_mean_ were identified at t_0_ and t_1_ for each patient, indicated as Δ(t_0_) and Δ(t_1_), respectively. This corresponded to the most hypermetabolic tumor for SUV_mean_ and SUV_max_, the tumor with the largest volume for V, and the tumor with the most restricted diffusion for ADC_mean_. Different target lesions were possible in the pre- and post-therapy scans, and for different metrics.

#### Lymphoid tissue segmentation and metric extraction

Spleen and bone marrow were manually segmented using 3Dslicer, with separate delineations performed for FDG PET, T1-weighted LAVA flex water MR and b = 900 s/mm^2^ DWI. The aim was to segment normal tissue without tumor involvement. Focal disease was detected as a part of the tumor segmentation task. If diffuse infiltration was present, the affected data set was excluded from the spleen or bone marrow assessment, as applicable.

The whole spleen was segmented. Focal disease was included in the spleen volume measurement, but excluded from the SUV_mean_ and ADC_mean_ measurements. Diffuse tumour infiltration was deemed present if the spleen SUV_mean_ (excluding focal disease) was higher than the SUV_mean_ of a 3 cm circular liver reference region [31]. For bone marrow, volumes of interest for vertebral bodies were drawn to include as much as possible of the tissue while excluding tissue borders and avoiding partial volume effects. Vertebral bodies in the lumbar spine were preferentially segmented (L1-L5), but if radiotherapy to the lumbar spine was included as bridging therapy the vertebral bodies of the thoracic spine was segmented instead (T8-T12). A vertebral body was excluded if focal disease was observed. If bone marrow SUV_mean_ was larger than the SUV_mean_ of the liver reference region, diffuse infiltration of bone marrow was deemed present [33].

Extracted metrics for the spleen were SUV_mean_, V and ADC_mean_, and for the bone marrow SUV_mean_, mean FF (FF_mean_) and ADC_mean_. Rates of change were calculated.

### Statistical analysis

Summary statistics are presented as the median and interquartile range (IQR) for continuous variables and as absolute values and percentages for categorical variables. Spearman’s correlation tested for associations between extracted metrics. Univariate Cox proportional hazards regression analysis identified predictive indicators for PFS and OS. In addition to extracted metrics, predictor variables also included the baseline metrics age, gender and BMI at t_0_, and Lugano classification at t_1_. For pre-therapy volume metrics (MTV, TLG, V and V_total_), the unit increase in the Cox regression analysis was changed from milliliter (ml) to deciliter (dl) for improved Hazard Ratio (HR) interpretability. For PFS, the endpoint was defined as relapse, progression, death from any cause or the time of last clinical follow-up, while for OS the endpoint was defined as death from any cause or the time of last clinical follow-up. For pre-therapy data evaluation, the starting time for PFS and OS was the date of the CAR T-cell infusion, while for post-therapy data evaluation, the starting time was set to t_1_. Time-to-event curves were produced using the Kaplan-Meier method for variables found statistically significant during the univariate analysis, with the difference in PFS and OS between subgroups assessed using a log-rank test. To dichotomize, median values were used. Uncorrected *p*-values < 0.05 were considered statistically significant. For the univariate Cox regression analysis, correction for multiple statistical testing was performed using the false discovery rate (FDR) [34], with FDR < 0.05 considered statistically significant. All statistical analyses were performed using the open-source R software (v3.6.1.).

## Results

### Patient characteristics

A total of 24 patients were included in the study (median age 63 years, range 14–76 years, 13 females), with CAR T-cell therapy given from November 2017 to January 2020. Of the 24 patients treated, measurable disease on imaging was not observed for 6 patients, an equipment failure occurred during pre-therapy PET/MR imaging for 1 patient, and due to MR contra-indications, 1 patient underwent PET/CT imaging. This left 16 patients eligible for further PET/MR evaluation (median age 63 years, range 37–71 years, 9 females), with baseline characteristics, infusion and imaging details, and clinical outcomes of therapy shown in Table 1.

**Table 1:** Baseline patient characteristics

Characteristic	Value
Age, median (range), y	63 (37–71)
Sex, female	9 (56)
BMI, median (range), kg/m^2^	27.7 (16.4–43.3)
**Histology**	
DLBCL	15 (94)
FL	1 (6)
**Treatment history**	
Number of prior lines, median (range)	4 (2–6)
Autologous transplant, yes	8 (50)
**Bridging therapy**	
Yes	14 (88)
Chemotherapy	11 (69)
Radiotherapy	3 (19)
**Number of CAR T-cell infusions**	
1	6 (38)
2	10 (63)
**Number of imaging sessions**	
2	7 (44)
3	8 (50)
> 3	1 (6)
**Lugano classification at t**_**1**_	
CMR	2 (13)
PMR	5 (31)
NMR	2 (13)
PMD	7 (44)
**Clinical follow-up**	
Follow-up survivors, median (range), mo	42.6 (36.0–48.2)
PFS from 1st infusion, median (IQR), mo	3.9 (1.8–7.8)
OS from 1st infusion, median (IQR), mo	9.3 (4.7–18.3)
PFS from t_1_, median (IQR), mo	3.2 (1.1–6.1)
OS from t_1_, median (IQR), mo	8.6 (4.0–17.6)

Baseline patient characteristics at the time of CAR T-cell infusion, infusion and imaging details and clinical outcomes of therapy. Values are presented as n (%) unless otherwise stated. *CMR* Complete Metabolic Response, *PMR* Partial Metabolic Response, *NMR* No Metabolic Response, *PMD* Progressive Metabolic Disease

During the follow-up time, progression was seen for 15 patients and death occurred for 13 patients. The pre-therapy scan was performed at a median time of 1 day (range 0–9 days) prior to CAR T-cell therapy. The majority of patients had pre-therapy imaging after lymphodepleting therapy (*n* = 15), but due to imaging scheduling issues one patient had lymphodepletion performed after imaging. The median time between CAR T-cell therapy and the first post-therapy scan was 3.1 weeks (range 2.9–4.0 weeks). A second post-therapy scan was performed for nine patients, at a median time of 8.9 weeks (range 7.0–16.1 weeks) after CAR T-cell infusion. One patient had four post-therapy scans, with the third and fourth scan at 4.6 and 6.0 months post-therapy, respectively.

### Extracted metrics

**Fig. 1:**
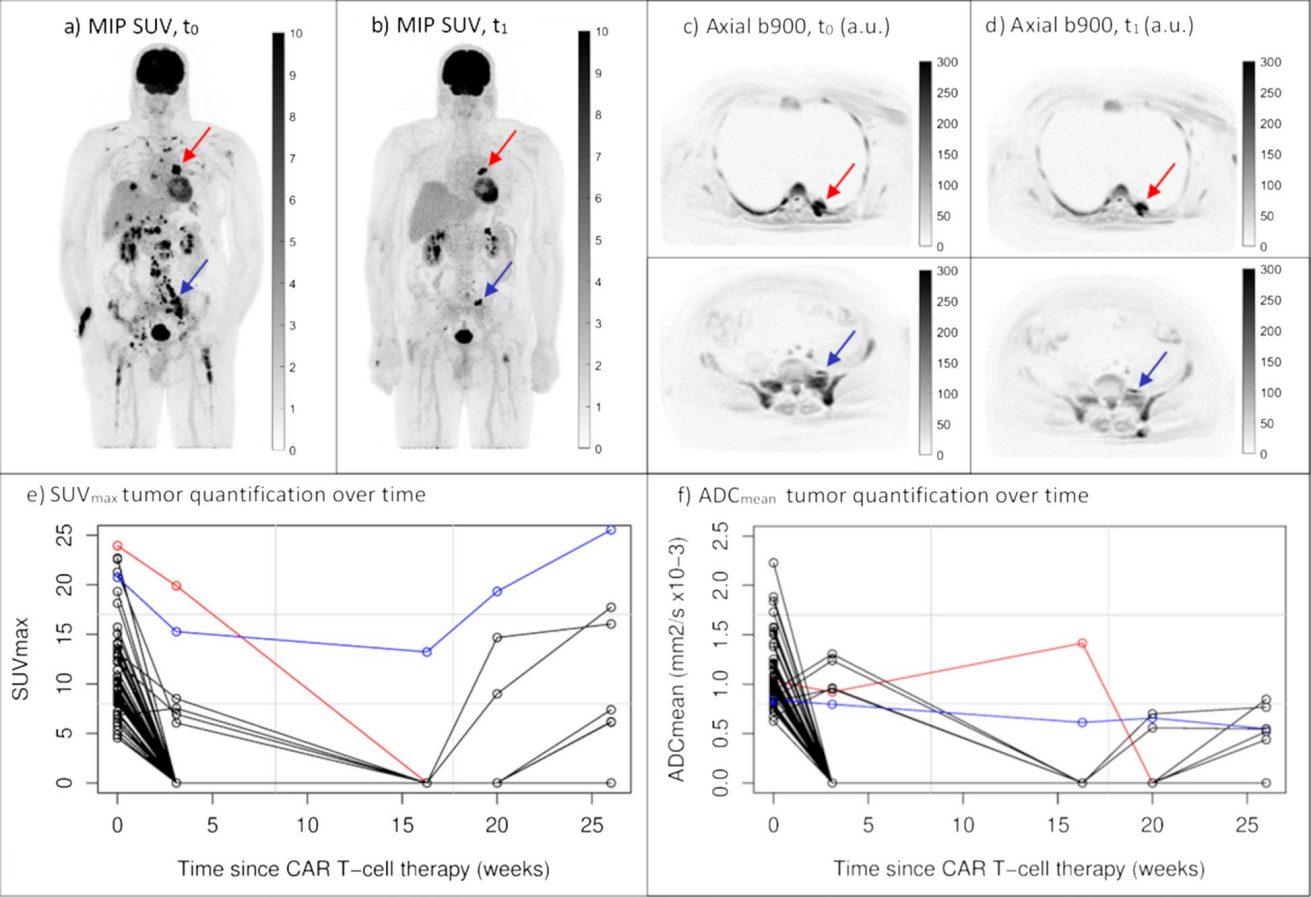
Example patient scanned over an extended period of time. Maximum Intensity Projection (MIP) SUV images (**a**, **b**), axial b900 DW images (**c**, **d**) and line graphs of tumor SUV_max_ and ADC_mean_ quantification over time (**e**, **f**). Pre-therapy (t_0_) and early post-therapy (t_1_) images are shown in inverted grey scale. A large decrease in MTV between the pre-therapy (MTV = 337 ml) and early post-therapy (MTV = 19 ml) scans was measured, as visualized by the MIP SUV images (**a**, **b**). Although this patient had a large total tumor burden pre-therapy, the OS was long (48.2 months with last follow-up as end-point). SUV_max_ and ADC_mean_ tumor quantification over time (**e**, **f**), indicate an intra-tumor heterogenic response to the CAR T-cell therapy. Target lesion selection post-therapy is shown for SUV_max_ (red arrows) and ADC_mean_ (blue arrows). These tumors are also highlighted in the corresponding color in the line graphs (**e**, **f**)

Table 2 shows a summary of extracted tumor and lymphoid tissue metrics, while example images of one patient are shown in Fig. 1. The median number of segmented lesions per patient was 4 (IQR 1–6) pre-therapy and 3 (IQR 1–6) post-therapy. The choice of target lesion for SUV_mean_, SUV_max_ and ADC_mean_ had an impact on the rate of change calculation (Table 2). Target lesions identified on the pre-therapy scan gave median rates of change indicative of therapy response (Δ(t_0_): SUV_mean_ = 0.94, SUV_max_ = 0.76, ADC_mean_ = 1.17), i.e. a trend of a less aggressive disease post-therapy. The opposite was seen for target lesion identification based on the post-therapy scan (Δ(t_1_): SUV_mean_ = 1.08, SUV_max_ = 1.02, ADC_mean_ = 0.92).

**Table 2:** Tumor and lymphoid organ metrics for the pre-therapy scan (t_0_), post-therapy scan (t_1_) and rates of change (Δ)

	t_**0**_	t_**1**_	Δ(t_**0**_)	Δ(t_**1**_)
**Tumor**				
MTV, ml	39.5 (7.9–284.4)	30.6 (4.9–224.6)	1.35 (0.49–2.31)	
TLG, ml	308.9 (19.1–1802.1)	132.8 (15.2–2085.9)	1.35 (0.29–3.73)	
SUV_mean_	7.6 (5.0–9.7)	6.2 (3.4–10.8)	0.94 (0.61–1.13)	1.08 (0.81–1.36)
SUV_max_	16.9 (9.9–24.6)	11.6 (6.9–25.9)	0.76 (0.53–1.42)	1.02 (0.67–1.53)
V, ml	27.2 (1.5–54.2)	12.9 (2.7–129.5)	1.37 (0.71–2.09)	1.37 (0.71–2.09)
V_total_, ml	34.7 (4.0–219.9)	17.6 (3.4–202.7)	1.60 (0.78–2.39)	
ADC_mean_, × 10^-3^ mm^2^/s	0.89 (0.66–1.10)	1.01 (0.71–1.34)	1.17 (1.03–1.64)	0.92 (0.80–1.16)
**Bone marrow**				
SUV_mean_	1.8 (1.2–2.4)	1.3 (0.9–1.9)	0.79 (0.66–0.86)	
FF, %	81 (73–88)	82 (79–86)	1.01 (0.99–1.09)	
ADC_mean_, × 10^-3^ mm^2^/s	0.49 (0.44–0.59)	0.51 (0.40–0.57)	1.01 (0.79–1.09)	
**Spleen**				
SUV_mean_	1.9 (1.7–2.2)	1.8 (1.5–2.1)	0.93 (0.79–0.99)	
V, ml	136.9 (83.1–273.7)	155.8 (93.1–261.7)	1.09 (0.99–1.21)	
ADC_mean_, × 10^-3^ mm^2^/s	1.04 (0.82–1.15)	0.91 (0.68–0.96)	0.91 (0.76–1.10)	

Median values with the IQR in parentheses. Δ(t_0_) corresponds to the rate of change with pre-therapy target lesion selection, while Δ(t_1_) corresponds to the rate of change with post-therapy target lesion selection

Example bone marrow segmentations of the lumbar spine are shown as Additional file 1. Bone marrow was segmented at the lumbar spine for 15 patients (15/16). Of these, due to focal tumor disease, the number of lumbar vertebras segmented were four in 14 patients and two in 1 patient. The thoracic spine (T11 and T12) was segmented in one patient, due to radiotherapy to the lumbar spine as part of the bridging therapy. Focal disease in the spleen was observed for one patient. Post-therapeutic bone marrow and spleen measurements were excluded in one patient because of large increases in FDG uptake of the whole bone marrow and spleen at t_1_, indicating diffuse infiltration. Diffuse FDG uptake in the bone marrow and spleen were not observed in any other patient (SUV_mean_ < liver SUV_mean_).

Several of the extracted metrics were strongly correlated with each other (ρ > 0.6, Additional file 2). Pre-therapy SUV and volume metrics were correlated (ρ = 0.80–0.99), while pre-therapy ADC_mean_ and volume metrics were negatively correlated (ρ = − 0.63-(− 0.70)). Rate of change metrics showed the same trend; Δ SUV and volume metrics were correlated (ρ = 0.61–0.98), while Δ(t_1_) ADC_mean_ was negatively correlated to Δ(t_1_) SUV_max_ (ρ = − 0.65), Δ(t_1_) SUV_mean_ (ρ = − 0.69) and Δ TLG (ρ = − 0.61).

Lugano classification at t_1_ was correlated with ΔSUV metrics (ρ = 0.71–0.81). In general, pre-therapy tumor metrics were not correlated with rate of change tumor metrics. The exception being a negative correlation for ADC_mean_ pre-therapy and ADC_mean_ Δ(t_0_) (ρ = − 0.61). Negative correlations between pre-therapy and rate of change metrics were also seen for lymphoid tissue, i.e. bone marrow FF_mean_ (ρ = − 0.82) and spleen ADC_mean_ (ρ = − 0.69). In addition, pre-therapy bone marrow and spleen SUV_mean_ were both correlated to the tumor Δ(t_1_) ADC_mean_ (ρ =0.65 and 0.72, respectively).

### Survival analysis

**Fig. 2:**
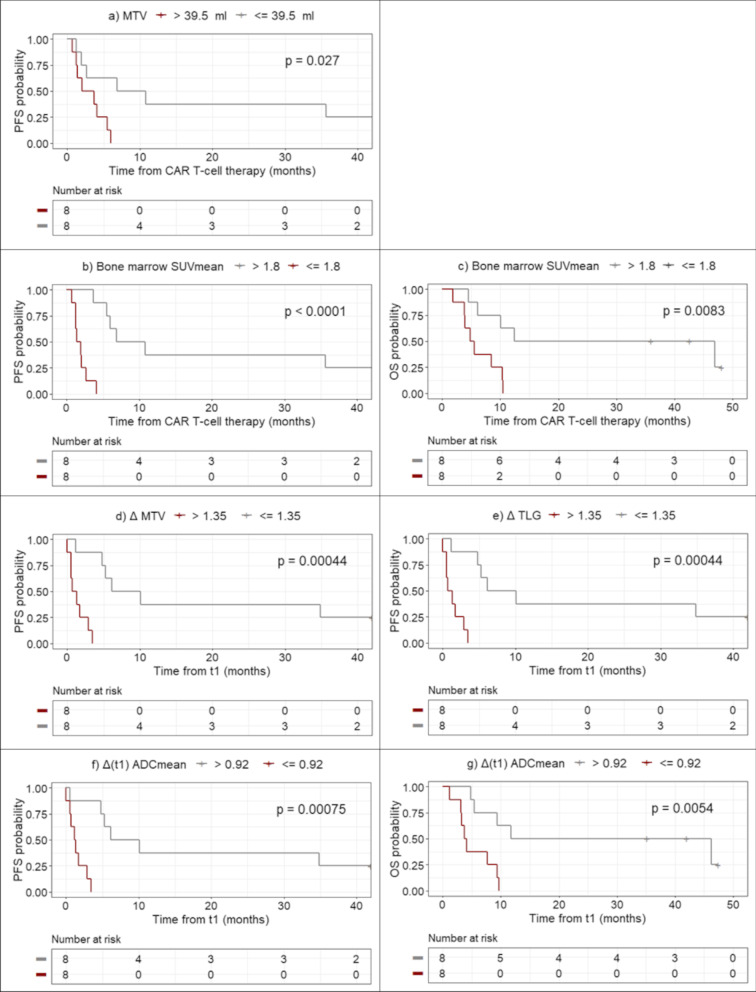
Kaplan-Meier survival curves and log-rank *p*-values for extracted tumor and bone marrow metrics. The median for each metric was used for thresholding. Pre-therapy results (top), showing MTV (**a**), and bone marrow SUV_mean_ (**b**,**c**). Post-therapy rate of change results (bottom) showing ΔMTV (**d**), ΔTLG (**e**), and Δ(t_1_) ADC_mean_ (**f**, **g**)

**Fig. 3:**
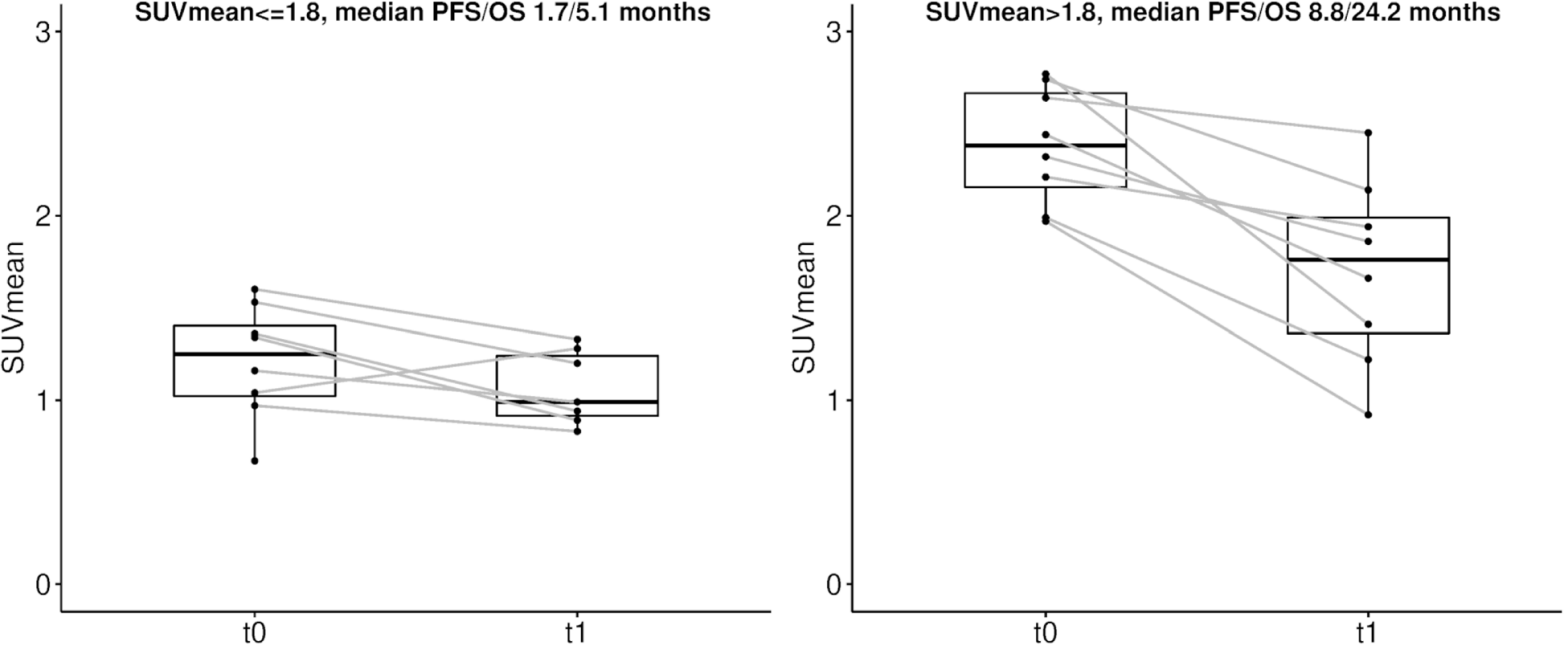
Individual bone marrow SUV_mean_ changes between the pre-therapy and 3-week post-therapy scan evaluations. Patients are split according to the median pre-therapy SUV_mean_ of 1.8., with longer PFS and OS observed for patients with higher pre-therapy bone marrow SUV_mean_. In general, the bone marrow SUV_mean_ decreased post-therapy

The results of the univariate Cox regression analysis for PFS and OS are shown in Table 3. According to uncorrected *p*-values, statistically significant metrics were further evaluated using the Kaplan-Meier method (Fig. 2). Individual bone marrow SUV_mean_ changes between the pre-therapy and 3-week post-therapy scan evaluations are shown in Fig. 3.

**Table 3:** Univariate analysis for PFS and OS

		Pre-therapy						Δ					
		PFS			OS			PFS			OS		
	Variable	HR (95% CI)	*p*-value	FDR	HR (95% CI)	*p*-value	FDR	HR (95% CI)	*p*-value	FDR	HR (95% CI)	*p*-value	FDR
Basic	Age	1.02 (0.95–1.11)	0.55	0.69	1.05 (0.97–1.14)	0.21	0.37	–	–	–	–	–	–
	BMI	0.89 (0.79–1.00)	0.051	0.15	0.96 (0.88–1.04)	0.29	0.41	–	–	–	–	–	–
	Gender	1.12 (0.37–3.34)	0.84	0.84	1.14 (0.36–3.62)	0.83	0.83	–	–	–	–	–	–
Lugano	2 groups	–	–		–	–		1.79 (0.60–5.39)	0.29	0.51	1.19 (0.37–3.76)	0.77	0.92
Tumor	Lesions	1.01 (0.97–1.05)	0.622	0.70	0.97 (0.92–1.03)	0.27	0.41	1.44 (0.66–3.16)	0.36	0.57	1.69 (0.78–3.69)	0.18	0.37
	MTV	1.48 (1.08–2.01)	**0.00079**	**0.012**	1.27 (0.98–1.65)	**0.046**	0.19	1.71 (1.11–2.64)	**0.0093**	0.070	1.40 (0.98–2.00)	0.058	0.19
	TLG	1.02 (1.00–1.04)	**0.0054**	**0.023**	1.03 (1.01–1.05)	**0.0014**	**0.024**	1.46 (1.03–2.05)	**0.022**	0.089	1.30 (0.97–1.75)	0.071	0.19
	V_total_	1.63 (1.14–2.34)	**0.0015**	**0.012**	1.34 (0.96–1.88)	0.075	0.19	1.95 (1.06–3.58)	**0.024**	0.089	1.74 (1.03–2.92)	**0.031**	0.19
	SUV_mean_, t_0_	1.06 (0.94–1.18)	0.35	0.54	1.10 (0.96–1.25)	0.18	0.37	1.34 (0.45–4.02)	0.60	0.75	1.06 (0.32–3.56)	0.93	0.98
	SUV_max_, t_0_	1.03 (0.99–1.06)	0.15	0.32	1.04 (0.99–1.08)	0.077	0.19	1.84 (0.87–3.90)	0.10	0.23	1.05 (0.61–1.83)	0.86	0.96
	V, t_0_	1.61 (1.06–2.43)	**0.013**	**0.043**	1.90 (1.14–3.19)	**0.0043**	**0.037**	1.63 (0.96–2.76)	0.062	0.20	1.45 (0.94–2.25)	0.086	0.20
	ADC_mean_, t_0_	0.82 (0.14–4.93)	0.83	0.84	1.28 (0.29–5.69)	0.75	0.79	0.69 (0.16–3.09)	0.63	0.75	0.44 (0.096–2.03)	0.29	0.46
	SUV_mean_, t_1_	–	–		–	–	–	2.12 (0.73–6.19)	0.17	0.31	1.91 (0.58–6.28)	0.28	0.46
	SUV_max_, t_1_	–	–		–	–	–	2.34 (1.17–4.71)	**0.011**	0.070	1.21 (0.75–1.96)	0.43	0.58
	V, t_1_	–	–		–	–	–	1.07 (0.98–1.16)	0.11	0.23	1.09 (0.99–1.19)	**0.038**	0.19
	ADC_mean_, t_1_	–	–		–	–	–	0.083 (0.012–0.57)	**0.0091**	0.070	0.099 (0.0099–0.99)	**0.040**	0.19
Bone marrow	SUV_mean_	0.24 (0.083–0.69)	**0.0044**	**0.023**	0.28 (0.094–0.83)	**0.014**	0.080	10.51 (0.77–143.40)	0.073	0.20	19.74 (0.77–504.00)	0.066	0.19
	FF_mean_	1.01 (0.97–1.06)	0.51	0.69	1.06 (0.99–1.14)	0.066	0.79	0.053 (0–95.21)	0.44	0.60	0 (0–1.97)	0.058	0.19
	ADC_mean_	3.54 (0.047–264.10)	0.57	0.69	0.44 (0.0051–37.28)	0.71	0.19	1.42 (0.070–28.76)	0.82	0.86	3.04 (0.14–65.09)	0.48	0.60
Spleen	SUV_mean_	0.24 (0.051–1.12)	0.066	0.16	0.65 (0.17–2.45)	0.52	0.68	1.42 (0.028–71.07)	0.86	0.86	7.32 (0.12–462.50)	0.35	0.50
	V	0.76 (0.45–1.29)	0.31	0.52	1.11 (0.71–1.72)	0.65	0.79	0.25 (0.0083–7.49)	0.42	0.60	0.15 (0.0061–3.51)	0.23	0.44
	ADC_mean_	0.27 (0.023–3.14)	0.29	0.52	0.26 (0.029–2.27)	0.22	0.37	1.76 (0.092–33.37)	0.71	0.79	0.97 (0.049–19.05)	0.98	0.98

Statistically significant results are depicted in bold (*p* < 0.05, FDR < 0.05). For rate of change (Δ) in tumor SUV_mean_, SUV_max_, V and ADC_mean_, the target lesion was selected at t_0_ and at t_1_. Hazard ratios (HRs) for volume metrics (MTV, TLG, V and V_total_) presented in dl unit change

None of the baseline metrics (age, gender and BMI) were associated with PFS or OS, neither was the Lugano classification at t_1_ (HR = 1.79 and *p* = 0.29 for PFS, HR = 1.19 and *p* = 0.77 for OS). The Lugano classification was grouped into responders (CMR + PMR, *n* = 7) and non-responders (NMR + PMD, *n* = 9) for the univariate Cox regression.

#### Tumor metrics, pre-therapy univariate analysis

For pre-therapy data, all volume metrics were significantly associated with PFS: MTV (HR = 1.48, p *p* < 0.001), TLG (HR = 1.02, *p* = 0.0054), V_total_ (HR = 1.63, *p* = 0.0015) and V (HR = 1.61, *p* = 0.013). Association with OS for pre-therapy metrics was seen for MTV (HR = 1.27, *p* = 0.046), TLG (HR = 1.03, p < 0.001) and V (HR = 1.90, *p* = 0.0043). These findings remained significant after correcting for multiple comparisons (FDR < 0.05), with the exception of pre-therapy MTV and OS (FDR = 0.19). Larger tumor volumes and/or higher tumor FDG uptake were associated with a shorter survival as measured by PFS or OS.

Kaplan Meier curves were produced, with the pre-therapy median MTV (≤39.5 ml) being the only measure able to separate patients into two groups using PFS as outcome measure (*p* = 0.027, Fig. 2a).

#### Tumor metrics, rate of change univariate analysis

Structural and metabolic total tumor burden rate of change were associated with PFS for ΔMTV (HR = 1.71, *p* = 0.0093), ΔTLG (HR = 1.46, *p* = 0.022) and ΔV_total_ (HR = 1.95, *p* = 0.024), and with OS for ΔV_total_ (HR = 1.74, *p* = 0.031). For these metrics, a larger increase post-therapy was associated with shorter PFS or OS. For ΔSUV_max_, ΔV and ΔADC_mean_, the target lesion identification had to be performed post-therapy for significant association with PFS and/or OS. Δ(t_1_) SUV_max_ was associated with PFS (HR = 2.34, *p* = 0.011) and Δ(t_1_) V was associated with OS (HR = 1.09, *p* = 0.038). A larger increase in tumor metabolism and a larger increase in tumor volume were associated with shorter PFS and OS, respectively. Δ(t_1_) ADC_mean_ was associated with both PFS (HR = 0.083, *p* = 0.0091) and OS (HR = 0.099, *p* = 0.040), with a larger decrease in ADC_mean_ post-therapy associated with shorter PFS and OS. None of the tumor rate of change metrics remained statistically significant after correction for multiple comparisons (FDR > 0.05).

For the Kaplan Meier analysis, the median ΔMTV (≤1.35) and ΔTLG (≤1.35) were able to separate patients into two groups according to PFS (*p* < 0.001 for both) (Fig. 2d, e), while median Δ(t_1_) ADC_mean_ (≤0.92) was able to separate patients into two groups according to both PFS (p < 0.001) and OS (*p* = 0.0054) (Fig. 2f, g).

#### Lymphoid tissue metrics, pre-therapy univariate analysis

Of the pre-therapy lymphoid tissue metrics, bone marrow SUV_mean_ was associated with therapy outcome (Table 3). Pre-therapy bone marrow SUV_mean_ was associated with both PFS (HR = 0.24, *p* = 0.0044) and OS (HR = 0.28, *p* = 0.014), with a higher bone marrow FDG uptake pre-therapy corresponding to longer PFS and OS. After correcting for multiple comparisons, association with PFS remained statistically significant (FDR = 0.023), while association with OS did not (FDR = 0.080). By thresholding on the median SUV_mean_ (≤1.8), bone marrow FDG uptake was able to separate patients into two groups according to both PFS (*p* < 0.001) and OS (*p* = 0.0083) (Figs. 2b, c, 3).

For patients with pre-therapy bone marrow SUV_mean_ > 1.8, five patients received chemotherapy and one patient received radiotherapy as bridging therapy, two patients did not receive bridging therapy. For patients with pre-therapy bone marrow SUV_mean_ ≤ 1.8, six patients received chemotherapy and two patients received radiotherapy as bridging therapy. There was no correlation between type of bridging therapy and pre-therapy bone marrow SUV_mean_ (ρ = 0.03).

#### Lymphoid tissue metrics, rate of change univariate analysis

For lymphoid tissue rate of change, no metrics were associated with PFS or OS.

## Discussion

This study showed that whole body FDG PET/MR with DWI is a promising tool for predicting CAR T-cell therapy response in patients with r/r LBCL, with the total metabolic tumor burden, tumor ADC_mean_ and FDG uptake in bone marrow unaffected by tumor infiltration being possible PET/MR parameters for prediction of PFS and OS.

In line with other imaging studies assessing predictive factors of progression after CAR T-cell therapy in LBCL [10, 11, 14, 15], the pre-therapy metabolic tumor burden was associated with outcome. A lower total tumor burden indicated longer PFS and OS. MTV, TLG and V_total_ were all statistically significant in the univariate Cox regression, but patient stratification was only possible using the MTV (median MTV ≤ 39.5 ml). Notable from Fig. 1 however, is that a high tumor burden pre-therapy does not necessarily mean a poor prognosis, as also noted by Dean et al. [10]. It is likely that a combination of imaging metrics and other biomarkers will give a more optimal prediction as described by e.g. Vercellino et al. [11], identifying lymphoma burden (MTV and lactate dehydrogenase) and extranodal involvement as risk factors for early progression after CAR T-cell therapy. Previously published thresholds for patient stratification according to MTV vary substantially, e.g. 25 ml in [14] and 147.5 ml in [10]. This is most likely due to patient heterogeneity in the relatively small cohorts studied, differences in timing of imaging (i.e. imaging before or after bridging and/or lymphodepleting therapies) and choice of tumor segmentation method.

Rates of change for total tumor burden metrics were associated with PFS according to uncorrected *p*-values, but not when correcting for multiple comparisons. Patient stratification was possible for ΔMTV and ΔTLG (Δ ≤ 1.35 for both). As expected, larger post-therapeutic increases in the total tumor burden were associated with poorer survival. ΔSUV_max_ and ΔADC_mean_ were associated with survival if the target lesion was selected post-therapy and for uncorrected *p*-values (FDR > 0.05). Pre-therapy target lesion identification was not associated with PFS or OS, and neither was Lugano classification. To predict treatment response using ΔSUV_max_ and ΔADC_mean_, these results suggest that the most resistant lesion has to be identified post-therapy. In this heavily treated patient group of r/r LBCL the intra-patient tumor heterogeneity is likely to be large. Indeed, an intra-patient heterogenic response to CAR T-cell therapy was visible in patients with extended follow-up, as exemplified in Fig. 1.

Imaging studies assessing outcome prediction of CAR T-cell therapy in LBCL mainly use PET/CT scanners. The results of the present study show that PET/MR imaging can be a potential alternative. As well as providing the established benefits of FDG PET semi-quantification, this hybrid imaging modality gives the advantage of tumor ADC quantification. Due to their high cellularity and high nuclear-to-cytoplasm ratio, pre-therapy lymphoma lesions exhibit low ADC values [20]. Post-therapy, it has been suggested that response-induced cell swelling and apoptosis occur, causing an increase in tumor ADC as a sign of therapy response [21]. A link between increased ADC-values post-therapy and a favorable treatment outcome has been reported for non-Hodgkin lymphoma patients undergoing chemo- and immunochemo-therapy [22–24]. This is in line with the results of the current study, indicating that increased tumor ADC-values post-therapy are predictive of longer PFS and OS. Moreover, the median ADC_mean_ rate of change measured for the cohort (Δ(t_1_) ≤ 0.92) allowed for patient stratification.

The tumor FDG metrics Δ(t_1_)MTV, Δ(t_1_)TLG and Δ(t_1_)SUV_max_ were all associated with PFS for uncorrected *p*-values, but unlike the tumor Δ(t_1_) ADC_mean_ no association with OS was seen. This might indicate that the ADC is a more sensitive imaging biomarker post-therapy. One reason could be that FDG is a non-specific tracer in the sense that inflammatory changes and increased tumor metabolism are indistinguishable in the PET images [35]. Inflammatory changes post-therapy give rise to an influx of inflammatory cells, which would decrease the tumor ADC. This effect is however counteracted by oedema, cell membrane deterioration and apoptosis, giving a net effect of stable or increased tumor ADC-values for responding tumors. This is the opposite from the lowered ADC-values seen for truly progressing tumors [36]. In line with this finding, ADC has been reported to show early tumor changes in immunotherapy of glioblastoma [37, 38] and malignant melanoma [39].

ADC_mean_ is a widely used global summary statistic of whole body DWI. There are however a large number of additional DWI metrics that could be explored, including the minimum ADC, histogram analysis (kurtosis, skewness and percentiles) and intravoxel incoherent motion, that have shown promise in various cancer applications [40]. Although this study shows preliminary findings of whole body DWI being a promising tool for very early response assessment in patients with LBCL undergoing CAR T-cell therapy, larger cohort studies using additional MR parameters are needed to fully understand the potential of PET/MRI in this context.

Lymphoid tissue assessments showed that higher pre-therapy SUV_mean_ in the bone marrow was associated with longer PFS (*p* = 0.0044, FDR = 0.023) and OS (*p* = 0.014, FDR = 0.080), and patient stratification based on this parameter was possible according to the Kaplan-Meier analysis (median SUV_mean_ ≤ 1.8). Interestingly, the opposite results have been reported for other cancer types treated with conventional therapies. It was published that in breast, colorectal and stomach cancer, increased pre-therapy bone marrow FDG uptake has been linked to poor survival [41–44]. For CAR T-cell therapy in lymphoma, a potential explanation is that elevated pre-therapy FDG uptake might indicate bone marrow hyperactivity as a part of a systemic immune response. This is a complex process which includes activation of bone marrow and release of chemokines and cytokines [45]. It is known that chemokines are characterised as having a dual impact on cancer [46, 47]. Chemokines induce inflammatory changes in tissues and promote tumor angiogenesis, formation of metastatic niches and cancer cell growth [48], as such promoting tumor growth and development of distant metastases. This is also known as the oncological hypothesis of metastatic growth named “seed and soil” [49, 50] suggesting that a metastatic niche is likely to appear in the proper tissue environment. On the other hand, chemokines are able to activate and attract lymphocytes which can promote CAR T-cell expansion and effector functions [51, 52], contributing to better outcome. It has been reported that chemokines promote T-cells effector functions and direct migration of the immune cells in solid tumors [53, 54]. This study suggests that this stimulatory effect of chemokines is dominant in CAR T-cell therapy in LBCL and is in line with Hirayama et al. [55], who reported that patients with increased levels of specific chemokines and cytokines (MCP-1 and IL-7) before CAR T-cell treatment had better outcomes.

Our study includes limitations. The sample size was small (*n* = 16), meaning multivariable statistical analysis was not possible. Many of the extracted metrics were correlated, and should be interchangeable. This study should therefore be viewed as explorative and larger studies carried out to confirm the results. Due to the explorative nature of the study, we have reported both uncorrected *p*-values and FDRs. The results from the former should be interpreted with caution. The lymphoid tissue assessment required delineation of tissues unaffected by tumor infiltration. A limitation of the current study is that no bone marrow biopsies were performed, as this is the standard for assessment of bone marrow involvement. Recently, it has however been reported that FDG PET has high sensitivity and specificity for detection of bone marrow involvement in aggressive B-cell non-Hodgkin lymphoma, with a significantly higher sensitivity compared to bone marrow biopsy [33]. In addition, diffuse bone marrow infiltration is in general linked to an increased FDG uptake, and to a more aggressive disease and poorer prognosis. The results from the current study, with increased pre-therapy SUV_mean_ being associated with longer PFS and OS, further indicates that normal bone marrow was likely measured. Usage of PET/MR instead of PET/CT was also preferential in this regard, given the advantage of MR for visualizing metastatic infiltration [56]. We set the minimum lesion size for analysis according to Lugano classification (longest diameter > 15 cm for nodal disease and > 10 cm for extra-nodal disease). As a result, partial volume effects might affect the results, in particular for SUV_mean_ and ADC_mean_ measurements of small lesions. This could be one potential explanation for the tumor ΔSUV_max_ being associated with PFS, but not the ΔSUV_mean_. For clinical implementation, the total tumor burden metrics used in this study have the disadvantage of requiring manual input. Ongoing developments, in particular for automated FDG tumor segmentation, are however likely to substantially decrease the manual input needed, potentially making these measurements clinically feasible. Lastly, a limitation of the current study is that an investigational product was used for the CAR T-cell therapy.

## Conclusions

In r/r LBCL patients undergoing CAR T-cell therapy total metabolic tumor burden, tumor ADC_mean_ and FDG uptake in bone marrow unaffected by tumor infiltration are possible PET/MR parameters for prediction of PFS and OS. The findings from this explorative study suggest that PET/MR can be a feasible imaging modality for CAR T-cell therapy evaluation in LBCL, and that a combination of FDG PET/MR-derived imaging metrics may be useful for therapy outcome prediction.

## Supplementary Information

**Additional file 1.** Example bone marrow segmentations.

**Additional file 2.** Matrix showing the Spearman’s correlation coefficients (ρ) between extracted metrics.

## Data Availability

The datasets analysed during the current study are available from the corresponding author on reasonable request.

## Abbreviations

ADC_mean_: Mean Apparent Diffusion Coefficient
BMI: Body Mass Index
CAR: Chimeric Antigen Receptor
CIT: Checkpoint Inhibitor Therapy
CMR: Complete Metabolic Response
CT: Computed Tomography
DWI: Diffusion Weighted Imaging
FDG: F18-Fluorodeoxyglucose
FDR: False Discovery Rate
FF_mean_: Mean Fat Fraction
FOV: Field of View
HR: Hazard Ratio
LBCL: Large B-cell Lymphoma
MIP: Maximum Intensity Projection
MR: Magnetic Resonance
MTV: Metabolic Tumor Volume
NMR: No Metabolic Response
OS: Overall Survival
PET: Positron Emission Tomography
PFS: Progression Free Survival
PMD: Progressive Metabolic Disease
PMR: Partial Metabolic Response
Δ: Rate of Change
r/r: relapsed/refractory
SUV_max_: Maximum Standardized Uptake Value
SUV_mean_: Mean Standardized Uptake Value
TLG: Total Lesion Glycolysis
TE: Echo Time
TI: Inversion Time
TR: Repetition Time
V: Structural tumor volume
V_total_: Total structural tumor volume
