# Correction to: Whole body FDG PET/MR for progression free and overall survival prediction in patients with relapsed/refractory large B-cell lymphomas undergoing CAR T-cell therapy

**DOI:** 10.1186/s40644-023-00548-9

**Published:** 2023-05-19

**Authors:** Therese Sjöholm, Alexander Korenyushkin, Gustav Gammelgård, Tina Sarén, Tanja Lövgren, Angelica Loskog, Magnus Essand, Joel Kullberg, Gunilla Enblad, Håkan Ahlström

**Affiliations:** 1 Department of Surgical Sciences, Uppsala University, Uppsala, Sweden; 2 Antaros Medical AB, Mölndal, Sweden; 3 Department of Immunology, Genetics and Pathology, Uppsala University, Uppsala, Sweden

**Correction to:** ***Cancer Imaging*** **22** **, 76 (2022).**

10.1186/s40644-022-00513-y

During the publication process of the original article the arrows of Fig. 1 were misplaced. The correct figure is available in this correction article, the original article has been updated.

**Fig. 1:**
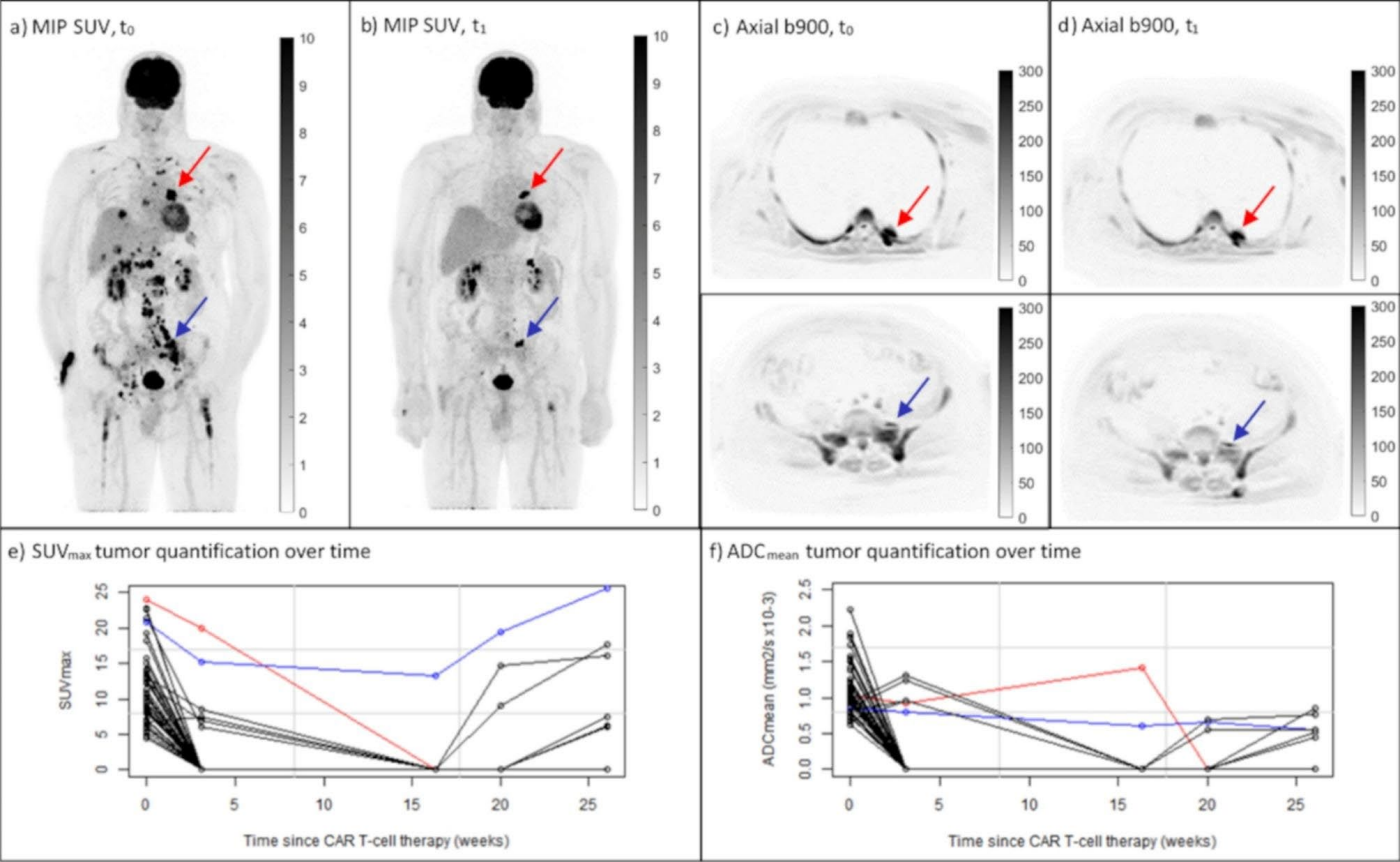
Example patient scanned over an extended period of time. Maximum Intensity Projection (MIP) SUV images (**a**, **b**), axial b900 DW images (**c**, **d**) and line graphs of tumor SUV_max_ and ADC_mean_ quantification over time (**e**, **f**). Pre-therapy (t_0_) and early post-therapy (t_1_) images are shown in inverted grey scale. A large decrease in MTV between the pre-therapy (MTV = 337 ml) and early post-therapy (MTV = 19 ml) scans was measured, as visualized by the MIP SUV images (**a**, **b**). Although this patient had a large total tumor burden pre-therapy, the OS was long (48.2 months with last follow-up as end-point). SUV_max_ and ADC_mean_ tumor quantification over time (**e**, **f**), indicate an intra-tumor heterogenic response to the CAR T-cell therapy. Target lesion selection post-therapy is shown for SUV_max_ (red arrows) and ADC_mean_ (blue arrows). These tumors are also highlighted in the corresponding color in the line graphs (**e**, **f**)

