# Supplementary material for: Whole body FDG PET/MR for progression free and overall survival prediction in patients with relapsed/refractory large B-cell lymphomas undergoing CAR T-cell therapy
Source: Cancer Imaging. 2022 Dec 27;22:76. doi: 10.1186/s40644-022-00513-y (PMC9793670; doi:10.1186/s40644-022-00513-y)

Additional file 1. Example of bone marrow segmentations for one representative subject for the water (a) and fat (b) structural MR, DWI b=900 s/mm^2 (c) and SUV FDG PET (d) data. Four vertebral bodies were segmented: L2-L5. The DWI and FDG PET images are displayed in inverted grey scale.

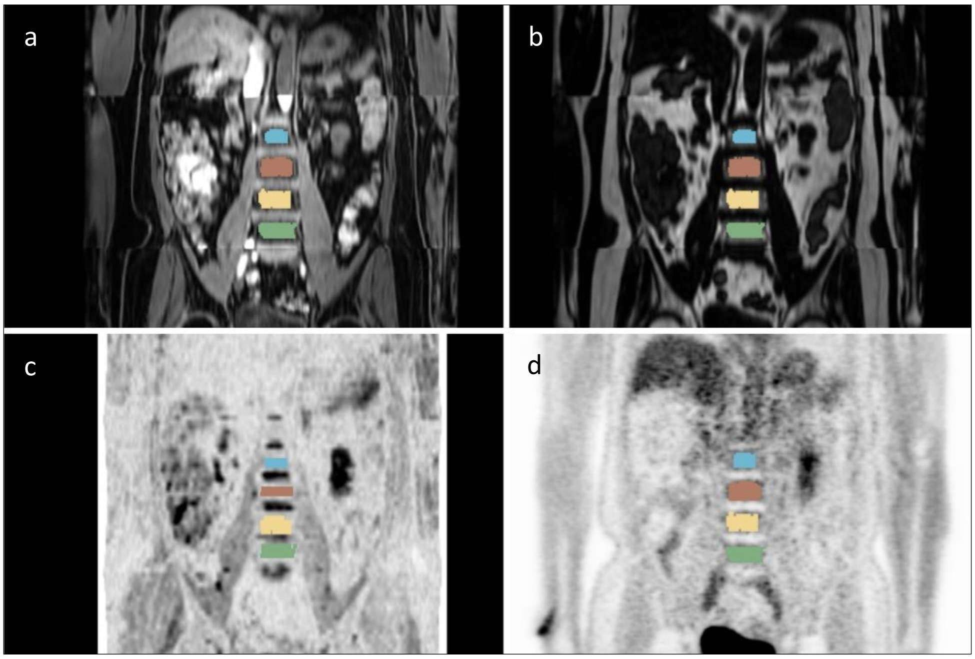

Supplement: Supplementary file 1 — Additional file 1. Example bone marrow segmentations. [file 40644_2022_513_MOESM1_ESM.pdf]
