# Supplementary material for: Whole body FDG PET/MR for progression free and overall survival prediction in patients with relapsed/refractory large B-cell lymphomas undergoing CAR T-cell therapy
Source: Cancer Imaging. 2022 Dec 27;22:76. doi: 10.1186/s40644-022-00513-y (PMC9793670; doi:10.1186/s40644-022-00513-y)

Additional file 2. Matrix showing the Spearman's correlation coefficients (ρ) between extracted metrics. Only statistically significant correlations are displayed ($p<0.05$).

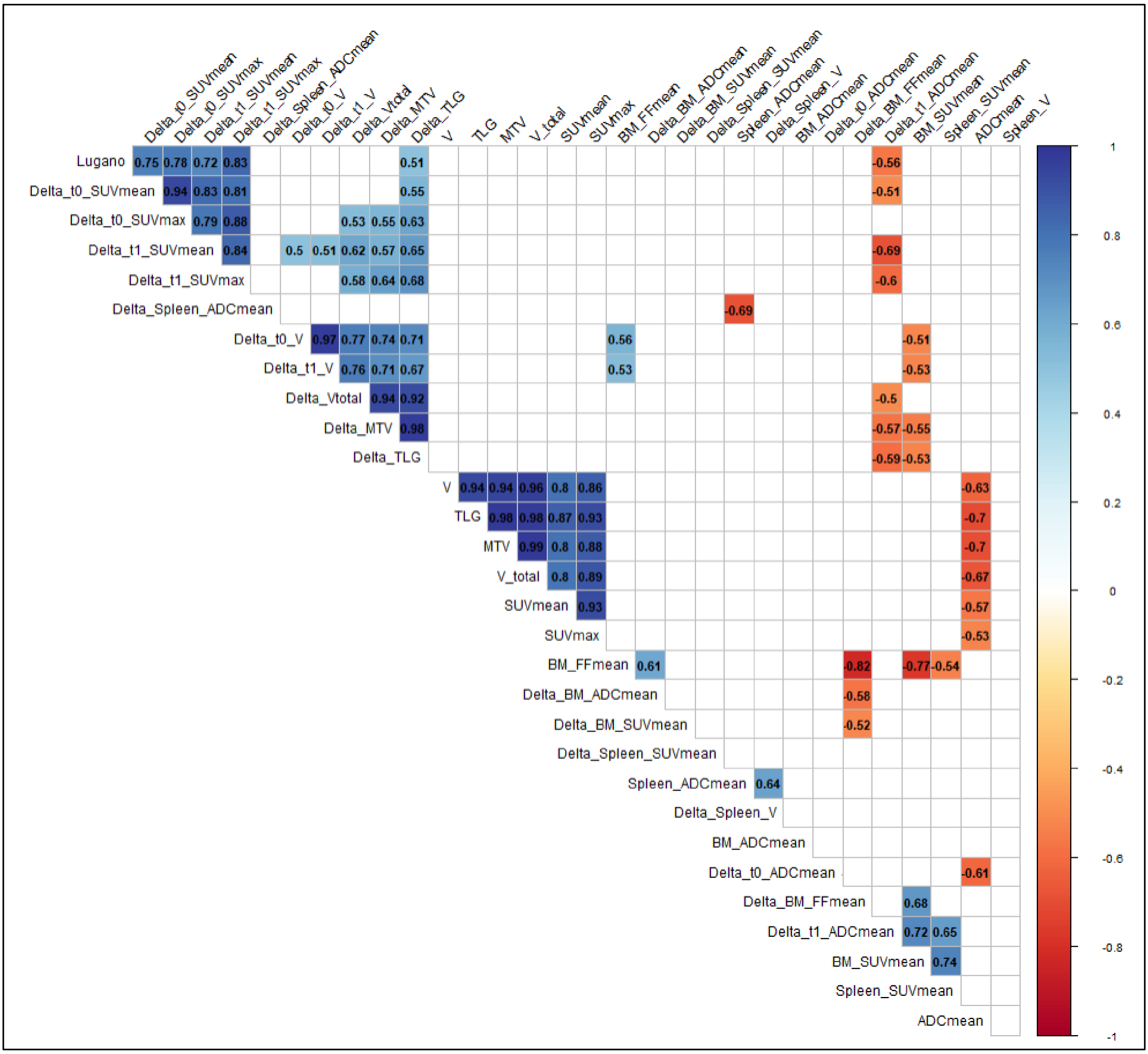

Supplement: Supplementary file 2 — Additional file 2. Matrix showing the Spearman’s correlation coefficients (ρ) between extracted metrics. [file 40644_2022_513_MOESM2_ESM.pdf]
